# Production of a Distilled Spirit Using Cassava Flour as Raw Material: Chemical Characterization and Sensory Profile

**DOI:** 10.3390/molecules25143228

**Published:** 2020-07-15

**Authors:** Eduardo Coelho, Lina F. Ballesteros, Lucília Domingues, Mar Vilanova, José A. Teixeira

**Affiliations:** 1 CEB–Centre of Biological Engineering, University of Minho, 4710-057 Braga, Portugal; e.coelho@ceb.uminho.pt (E.C.); linafernanda37@gmail.com (L.F.B.); luciliad@deb.uminho.pt (L.D.); 2 Misión Biológica de Galicia, Consejo Superior de Investigaciones Científicas (CSIC), El Palacio, Salcedo, 36143 Pontevedra, Spain

**Keywords:** cassava flour, enzymatic hydrolysis, alcoholic fermentation, distilled spirits

## Abstract

Cassava plays a key role in the food production and economies of several countries worldwide. Due to its starch content, alcoholic fermentation is a promising transformation process for adding value to cassava. However, most of the existing cassava beverages are from traditional origin, with the yields and quality often poorly known or controlled due to the use of artisanal production processes. This work aims at the application of easily implementable biotechnological tools for the production of cassava spirits, in order to add value to this raw material. Cassava flour was liquefied and saccharified using enzymatic cocktails, generating a fermentable broth with ~184 g L^−1^ of fermentable sugars. This was then fermented into an alcoholic product with ~10% ethanol by volume and distilled for spirit production. Cassava spirits with 40% ethanol by volume, with or without application of oak wood, were produced. For further valorization, volatile fractions of cassava spirits were characterized by gas chromatography–flame ionization detection (GC-FID) and GC–MS. These showed a predominance of yeast fermentation metabolites, complemented by wood extractives where oak chips were applied. Both produced spirits showed desirable sensory traits, receiving good acceptance by experienced tasters, demonstrating the feasibility of the proposed process to add value to cassava surplus.

## 1. Introduction

Cassava (*Manihot esculenta*) is a prominent food crop, widely known for feeding a significant fraction of the world’s population. This tuberous root, which is an excellent source of starch, grows on a wide variety of soils, and it is currently cultivated throughout tropical and subtropical regions worldwide [1]. By 2014, cassava production occurred in 103 countries, accounting for 270 million tons, and 25 million hectares cultivated worldwide, making it the 5^th^ most prominent staple crop [2]. This culture is particularly important in sub-Saharan African countries, which account for 50% of the total cassava production around the world, mainly due to its ease in cultivation and resistance to poor soils and dry weather [1,2]. Cassava can be used as a raw material for different purposes: it can be directly used as a food source, serve as livestock feed, or be transformed for production of energy or other commodities [3,4]. Its utilization strongly depends on the countries’ socio-economical context, being that research efforts continuously focus on increasing yields and adding value to cassava in order to tackle famine, poverty, and promote economic growth [5]. When cassava surplus is available, its transformation and production of commodities can be an advisable strategy to add value to this raw material, in opposition to price reduction [6]. 

One possibility is the conversion of cassava starch to ethanol, as the production of fermented alcoholic beverages is an attractive strategy for adding value to cassava surplus, suitable for developing countries, as it does not compete with food supply. Some alcoholic cassava beverages already exist, for example, cassava beer, traditionally consumed by indigenous people in the Amazon [7], as well as in India, where research efforts were recently performed to valorize local beer production from cassava [8]. Further cassava beverages can be mentioned, namely, cassava wines, for instance Parakari, popular among Guyana Amerindians, and Tapai, consumed among ethnic groups of East Malaysia [9]. Other examples of cassava-based alcoholic beverages can be mentioned, namely, distilled cassava spirits produced in indigenous communities in Cameroon where traditional methods are employed [10] or Tiquira, an artisanal spirit produced in the Maranhão State in Brazil [11]. Another case is found in Rwanda, where a distilled alcoholic spirit named Kanyanga is produced, often using cassava as raw material, also employing traditional methods [12]. However, traditional and uncontrolled production processes of Kanyanga can lead to toxic concentrations of congeners (isoamyl alcohol, isobutanol, and methanol), similarly to Kachasu, another traditional distilled beverage from Zambia, Zimbabwe, DR Congo, and Malawi, which led to prohibition Kanyanga distribution in Rwanda [12]. 

The application of traditional production processes and lack of knowledge regarding the final product mostly leads to low yield, low-quality beverages, or both, therefore poorly adding value to the raw material. Instead, biotechnological tools, often simple and easily implementable, can be used to produce cassava beverages. Several processes and tools are available for the production of ethanol from cassava starch, as seen for production of 1^st^ or 2^nd^ generation bioethanol, where the application of knowledge and technology allows achieving high yields [4,13]. Biotechnological tools such as enzymes for the hydrolysis of starch and robust yeast for ethanol production have already been applied to cassava with the aim on bioethanol production [14,15]. These tools can be shifted towards the production of alcoholic cassava beverages, in order to design controlled processes capable of generating quality added-value products. Furthermore, to the best of our knowledge, despite the studies reporting the production of cassava spirit drinks, there is not any study in the literature on the evaluation of chemical and sensorial characteristics of this type of beverage. Therefore, this work demonstrates a proof of concept on the production of a distilled spirit from cassava flour, applying biotechnological tools and processes. Ethanol titers and chemical and sensory characteristics of the produced beverages are presented, highlighting the use of cassava as a promising raw material to obtain a feasible and consumer-accepted distilled spirit beverage through easily implementable biotechnological tools. 

## 2. Results

### 2.1. Production of a Cassava-Based Fermentable Broth

**Figure 1:**
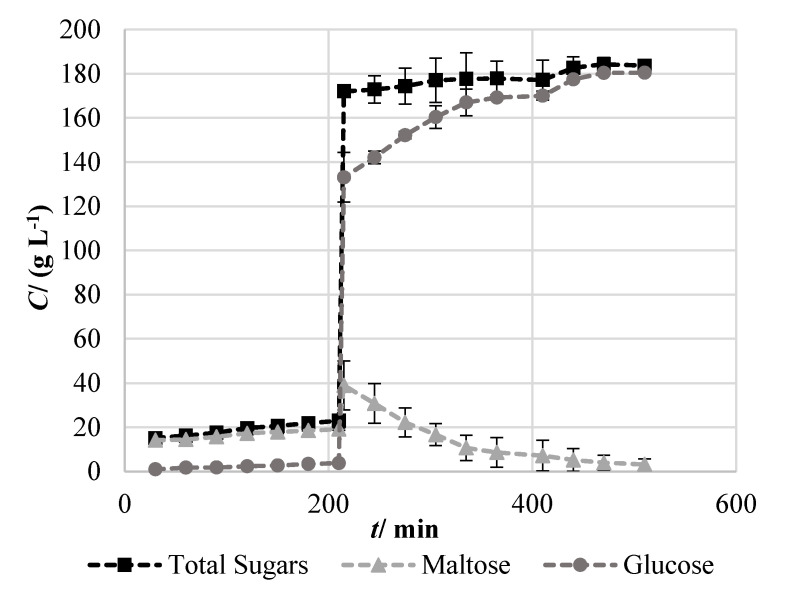
Profiling of fermentable sugar concentration (*C*) throughout time (*t*), during liquefaction and saccharification of cassava flour. Results express the mean ± standard deviation of independent samples (n = 2).

Considering the aim of producing a distilled spirit using cassava flour as raw material, the first step involved hydrolysis of cassava starch into fermentable sugars for alcoholic fermentation by yeast. As cassava flour is a dry raw material (with a moisture of ~11%), a process similar to the production of alcohol from grain was envisaged. However, cassava does not have the hydrolytic enzymatic activity typically found in malt, being that specific enzymes had to be supplemented for liquefaction and saccharification, mimicking a mashing step. Cassava flour has a starch content of 75 ± 1% of its dry weight, which serves as basis for determining the process yield and efficiency. A two-step enzymatic process was performed. First, a liquefaction step was carried out by the action of the Termamyl SC (Novozymes^®^, Bagsværd, Denmark), followed by a saccharification step for breaking the starch polymeric chain using the SAN Super 360L cocktail (Novozymes^®^, Bagsværd, Denmark). Profiling of starch hydrolysis was performed by quantification of maltose and glucose into the fermentation broth, presented in Figure 1. As visible in the results, during the liquefaction step a small increase in the concentrations of fermentable sugars occurred, probably due to the thermal degradation of polysaccharides. However, a more pronounced increase was verified after the addition of the SAN Super 360L cocktail for the saccharification step. Approximately 130 g L^−1^ of glucose was released after 5 min of enzymatic activity, followed by a less pronounced variation until reaching the final value of 184 g L^−1^. This second, less pronounced, increase in glucose concentration is mainly related to the breakage of maltose, visible by the decrease of its concentration. Moreover, additional hydrolysis of polysaccharides occurred, considering that 35 g L^−1^ of maltose was hydrolyzed but glucose concentration increased 47 g L^−1^. As a result, from 215 min until the end of the process, total sugar content increased in 12 g L^−1^, stabilizing around 440 min. After saccharification of cassava flour, a fermentable broth containing 184 g L^−1^ of glucose was obtained, accounting for a hydrolysis efficiency of 73% of the starch initially available in the raw material. This efficiency can be a target for further optimization, by selecting more suitable enzymes and process conditions.

In a similar approach, Ruiz and collaborators achieved an efficiency of ~80%, using an optimized enzymatic process for the hydrolysis of cassava starch [15]. Nevertheless, glucose concentrations obtained in this work are well within the sugar content typically found in wine musts, allowing the production of an alcoholic beverage with an ethanol concentration appropriate for distillation.

### 2.2. Fermentation and Distillation for Production of a Cassava Spirit

**Figure 2:**
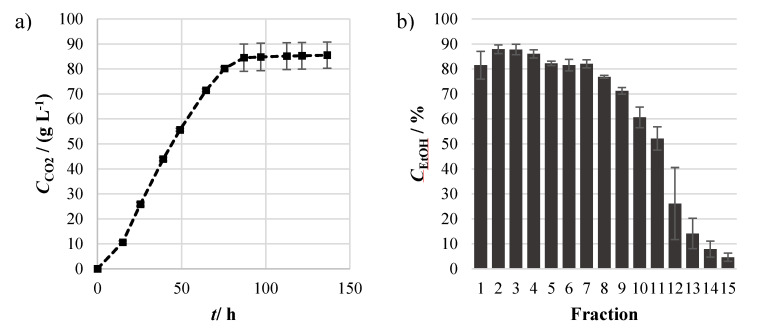
Profiling of (**a**) CO_2_ production (*C*_CO2_) during alcoholic fermentation of cassava flour broth throughout time (*t*) and (**b**) ethanol concentration (*C*_EtOH_) in the condensed fractions collected from distillation of fermented cassava broth. Results express the mean ± standard deviation of independent samples (n = 2).

Alcoholic fermentation of the cassava broth was carried out, accompanied by profiling of CO_2_ production, presented in Figure 2a. One important point to be highlighted is that cassava broth was generated solely from liquefied and saccharified cassava flour, and no additional nutritional supplementation, carbon, or nitrogen sources were supplied. As visible in the results, no difficulties in yeast start-up were observed, with the absence of a noticeable lag phase. CO_2_ production increased exponentially until ~87 h of fermentation, where the stationary state was attained. The fermented cassava product presented an ethanol concentration of 80.1 ± 3.0 g L^−1^, corresponding to a fermentation yield of 85% of the theoretical concerning total fermentable sugar concentration obtained from hydrolysis of starch, hinting an incomplete sugar to ethanol conversion by yeast. In fact, a residual content of 16.6 ± 8.0 g L^−1^ of fermentable sugars remained in the cassava fermented beverage. This “stuck” fermentation can be related to loss of yeast viability, product inhibition, or depletion of nitrogen source, which can also be potential targets for process optimization. Cumulative losses in saccharification and fermentation efficiencies led to an overall starch to ethanol conversion yield of ~61%. Slightly higher starch to ethanol conversion efficiency was achieved by Mayer and collaborators, reporting yields of ~77%. However, efficiencies obtained by Mayer and collaborators were achieved using lower substrate concentrations, therefore imposing lesser substrate inhibition and also leading to lower final ethanol titers, which can be disadvantageous for the subsequent distillation step [14]. After this, a secondary fermentation was carried out for the maturation of the alcoholic cassava product. Yeast was left in contact with the fermented broth during 60 days, for the assimilation and reduction of unwanted volatile compounds, such as diacetyl, which often causes an off-flavor in alcoholic beverages. During this secondary fermentation, ethanol concentration was maintained (79.5 ± 5.9 g L^−1^) and no additional conversion of sugars was observed (16.4 ± 9.2 g L^−1^), and an alcoholic fermented cassava product feasible for distillation was produced, with ethanol percentages by volume of about 10.0%.

These alcoholic products were posteriorly distilled for increasing ethanol concentration through fractional distillation. Several fractions were collected, and ethanol concentration was analyzed in each one as shown in Figure 2b. As visible, ethanol concentrations above 80% were observed in the initial seven fractions, after which a progressive drop was observed, reaching residual values of approximately 5% in the 15^th^ fraction. Almost all ethanol present in the fermented cassava was recovered in the collected fractions, with a distillation efficiency proximal to 100%. As a result, the production of undiluted cassava spirit with an ethanol concentration by volume of 62.4 ± 2.9% was possible. Later, ethanol concentration was rectified with ultrapure water to comply with the value commonly found in distilled spirits of 40%.

Finally, two distinct cassava spirit formulations were prepared. One of them was the original rectified spirit, without any further processing. The second formulation involved contact of the rectified spirit with oak chips. As widely acknowledged, wood aging is often associated with quality enhancement of distilled spirits, where the beverage extracts wood compounds that participate in its organoleptic quality. Thus, rapid induction of wood aroma was performed by contact of the rectified spirit with oak chips, based on previously reported observations [16].

### 2.3. Characterization of Cassava Spirits

Both cassava spirit formulations were characterized by gas chromatography–flame ionization detection (GC-FID) and GC–MS, for the quantification of volatile compounds occurring, respectively, at mg L^−1^ range and µg L^−1^ range. As observed in the results, several volatile compounds, namely, aldehydes, esters, alcohols, and acids, were identified and quantified in cassava spirits, which derive from yeast fermentative metabolism and can be further concentrated during distillation. Regarding major volatiles, presented in Table 1, several higher alcohols were found, accompanied by acetaldehyde and ethyl acetate, which are typical fermentation by-products and important compounds in spirit sensory quality. Higher alcohols 2-methyl-1-propanol, 2-methyl-1-butanol, and 3-methyl-1-butanol are important contributors to the sensory characteristics of distilled spirits, participating in the formation of the aromatic bouquet with solvent, “fusel-like”, and malty descriptors. Another important higher alcohol is 2-phenylethanol, which can contribute with a characteristic flowery rose-like aroma [17]. Higher alcohols derive from amino acids and sugars catabolism via the Ehrlich pathway and are common in fermented and distilled beverages [18]. Another commonly found compound in the fermented and distilled beverages is acetaldehyde, which was also found in the produced cassava spirits. Acetaldehyde contribution is strongly dependent on the produced beverages and the occurring concentrations. For instance, in vodka-type spirits, pungent unpleasant sensations are reported for acetaldehyde concentrations above 10 mg L^−1^, whereas in fruit or wine distillates, such unpleasant descriptors are not found in concentrations up to 100 mg L^−1^ [17]. Acetaldehyde was found in cassava spirits within intermediate values of the referred range, although no pungent or unpleasant descriptors were reported by the trained panelists.

Another important compound for distilled spirit quality is ethyl acetate, which was also found in cassava spirits within the interval reported for other distilled beverages such as whiskey, rum, and cachaça [19]. It is important to highlight that the overall concentration of congeners was well within the typical concentrations reported for distilled spirits [17], being that toxicity concerns regarding spirits produced by the presented method are avoided in opposition to the mentioned for traditionally produced spirits [12]. 

Regarding minor volatile compounds, presented in Table 2, cassava spirits showed several esters in their composition, with isoamyl acetate presenting the highest concentration, followed by ethyl hexanoate, and lastly by ethyl octanoate, 2-phenylethyl acetate, and ethyl hexadecanoate with similar concentrations. Particularly, isoamyl acetate, ethyl hexanoate, and ethyl octanoate concentrations largely exceeded the reported perception thresholds. When focusing on the application of wood, a decrease in the content of some esters was observed. For instance, the concentration of 2-phenylethyl acetate was lower in cassava spirit after contact with wood, which was even more prominent for ethyl hexadecanoate, showing a 6-fold decrease in its concentration. As reported, several compounds from the beverage can be adsorbed by wood, mainly driven by hydrophobic interactions, which can lead to a decreased content in esters, alcohols, or acids [16,20]. Nevertheless, in the presented results, only the two referred esters were found to adsorb in wood. Lactones were also found in relatively high concentrations, with γ-nonalactone quantified in both cassava spirits, at ~1000 µg L^−1^, and cis/trans-oak lactone detected only in cassava spirit with wood application. Lactones can derive from multiple sources, being typically associated with the degradation of lipids. For example, in cooperage processing, lactones derive from thermal degradation of wood lipids, being a very important compound in the sensory quality of aged beverages [21]. Thus, high concentrations of *cis*/*trans*-oak lactone were found in the cassava spirit with application of wood, which were extracted from oak chips by the hydroalcoholic matrix. Moreover, lactones can derive from microbial *de novo* production or transformation of fatty acids present in the raw material [22,23]. For example, lactones are commonly found in the fermentation of rice or when using it as an adjunct for brewing, resulting from biotransformation of lipids in the raw material [24,25,26]. Therefore, concentrations of lactones found in cassava spirits without application of wood can derive from specific composition of raw material, further modified by the fermentation process. Other distilled spirits also presented lactones in their composition, for example, American Bourbon whiskey where γ-nonalactone was the second most aroma-active compound found [27].

Volatile fatty acids, namely, hexanoic and octanoic acid, were found in cassava spirits, which also derive from yeast metabolism and are commonly associated with unwanted sensory defects. Nevertheless, these compounds were found in concentrations below their perception thresholds. The concentration of these compounds increased with application of wood, suggesting their extraction during contact of the cassava spirit with wood. Furfural was found in both formulations, whereas 5-methylfurfural was only found in cassava spirit with wood. Furan compounds usually derive from the degradation of polyosides: furfural can derive from the degradation of pentoses, for example, coming from hemicellulose, and 5-methylfurfural can derive from the degradation of glucose, either coming from cellulose or starch [21]. As visible in the results, cassava spirit without wood showed furfural content, hinting degradation of pentoses initially present in the raw material. Contact with wood led to further extraction of furan compounds, which are present in oak due to the thermal degradation of cellulose and hemicellulose [20,21]. 

**Figure 3:**
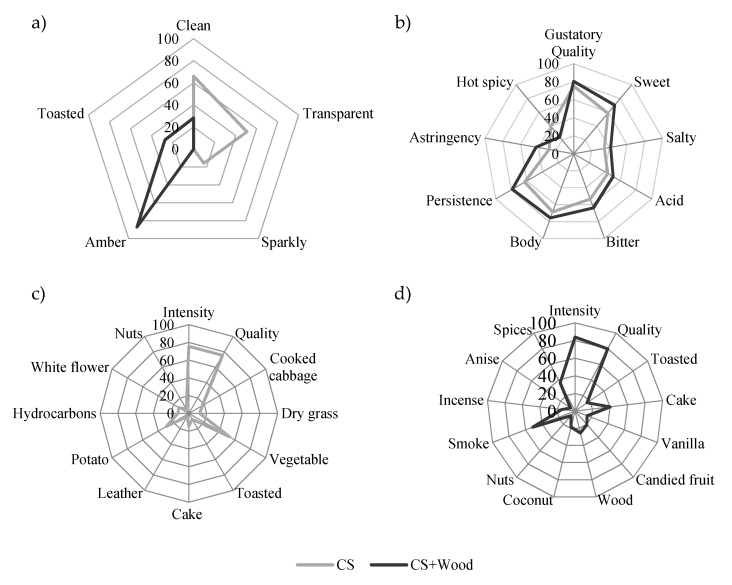
(**a**) Visual, (**b**) taste, and (**c**,**d**) aroma profiles in basis to geometric mean (GM%) of descriptors cassava spirit (CS) and cassava spirits with application of oak chips (CS+Wood).

Lastly, several compounds were exclusively found in cassava spirit with application of oak wood, namely, phenolic compounds and aldehydes. These are widely acknowledged by their influence of sensory properties of wood-aged beverages, and were extracted from oak chips by the hydroalcoholic matrix [28]. For example, concentrations of guaiacol and eugenol were higher than the reported perception thresholds, which led to the occurrence of smoky and spicy descriptors in the corresponding spirit, as visible in Figure 3d. Furthermore, vanilla concentration exceeded its perception threshold, which also led to its identification in CS+Wood.

For better perception of the characteristics and acceptability of the produced cassava spirits, a sensory characterization was performed by trained panelists, with results presented in Figure 3.

Regarding visual quality, cassava spirit without application of wood was described as transparent, clean, and sparkly, whereas after wood application, amber and toasted descriptors were predominant, as well as a less clear nature. Apart from inducing aroma compounds, wood application also transfers phenolic compounds that modified beverage pigmentation, leading to the amber and toasted features [29]. Focusing on gustatory descriptors, similar profiles were reported for both cassava spirit formulations and despite the slight differences observed; overall perceived gustatory quality was similar for both formulations. Cassava spirit without application of wood showed lower GM values for most of the gustatory descriptors, except for “hot spicy” sensation, for which it was higher. It is important to highlight that overall gustatory quality reached GM values of 80%, meaning a good acceptance by the trained panelists. This was also observed for perceived aroma quality, where both cassava spirit formulations presented GM scores of 80%, as well as intensities of 80%. Nevertheless, distinct aroma features were described for the two formulations. Cassava spirit without application of wood was marked by “vegetable” and “potato” descriptors, followed by “white flower” and “cake” aromas. “Cake” aroma was also found in cassava spirit with wood application, accompanied by “wood”, “smoke”, “coconut”, and “spices” descriptors, coherent with the previously described phenolic, lactone and aldehyde content.

It is important to highlight that a distilled spirit produced solely from cassava flour, showed good acceptance by experienced panelists, accustomed to tasting premium products protected by Geographic Indication. Moreover, performing a short contact with oak chips allowed the rapid induction of wood character, leading to a differentiated beverage with distinct sensory features but with similar acceptance and quality. With these results, cassava is shown as a suitable raw material for spirit production, providing a basis to foster the production of added-value products from this raw material.

## 3. Materials and Methods

### 3.1. Chemicals, Strains, and Raw Materials

The following compounds with the mentioned purities were used as standards for the GC analyses. From Fluka (St. Gallen, Switzerland): acetaldehyde (≥99.5%), 1-propanol (≥99.9%), 2-phenylethyl acetate (≥99%), 2-methyl-1-propanol (≥99.9%), 2-methyl-1-butanol (≥9%), 3-methyl-1-butanol (≥99.8%), 1-hexanol (≥99.9%), 2-phenylethanol (≥99%), hexanoic acid (≥98%), furfural (99%), and vanillin (≥98%). From Aldrich (Milwaukee, USA): methanol (≥99.8%), isoamyl acetate (≥99%), ethyl hexanoate (≥99%), ethyl octanoate (≥99%), diethyl succinate (99%), octanoic acid (≥99.5%), 5-methylfurfural (99%), cis/trans-oak lactone (≥98%), γ-nonalactone (≥98%), γ-decalactone (≥98%), guaiacol (9%), syringaldehyde (98%), eugenol (99%), and 2,6-dimethoxyphenol (99%). Ethyl hexadecanoate (≥99%) was purchased from Sigma, and ethyl acetate (99.8%) was purchased from Sigma-Aldrich. The remaining compounds were identified on the basis of NIST08 mass spectra library. Enzymes (Termamyl SC and SAN Super 360L) were obtained from Novozymes^®^, Copenhagen, Denmark. The yeast used for alcoholic fermentation was *Saccharomyces cerevisiae* (CA1185), which was isolated from cachaça fermentation processes, and obtained from UFLA (Federal University of Lavras, Lavras, Brazil) microbial collection. Cassava flour (with a moisture content of 10.8 ± 0.8) used in this work was supplied by the food company of Malange (Malange, Angola). The chemical composition of cassava flour consisted of (g/100 g) carbohydrates (86.4 ± 0.7, of which 75.5 ± 0.1 were starch, able to be transformed in fermented sugars), protein (0.8 ± 0.1), ashes (1.6 ± 0.1), and lipids (0.44 ± 0.2). 

### 3.2. Preparation of a Fermentable Cassava Broth

For generating a fermentable broth, cassava flour was hydrated, liquefied, and saccharified. In Schott flasks, a solution of 5 ppm CaCO_3_, pH 6, was heated up to 90 ± 5 °C using a magnetic stirrer with a heating plate and temperature probe (IKA C-MAG HS 7, Staufen, Germany). Termamyl SC enzyme (Novozymes^®^), used for the liquefaction stage, was added at a dosage of 1 g of enzyme per kg of cassava flour. Later, cassava flour at a concentration of 300 g L^−1^ was slowly added, and the mixture was maintained at constant temperature and agitation (90 ± 5 °C and 500 rpm, respectively). Liquefaction was carried out during 3.5 h with periodical sampling for sugar analysis. After that, rapid cooling of the broth to 55 °C was performed to enable saccharification stage, with a pH value of 6 confirmed. Saccharification was conducted by adding to the broth SAN Super 360L cocktail (Novozymes^®^), at a ratio of 1.8 mL per kg of cassava flour, maintaining the temperature between 55 and 60 °C, and using constant agitation (500 rpm). Periodical sampling was also performed for 5 h for accompanying hydrolysis profile by High-Performance Liquid Chromatography (HPLC). The liquefaction and saccharification processes were performed in duplicated in order to generate two independent fermentable cassava broths. 

### 3.3. Alcoholic Fermentation

Prior to alcoholic fermentation, saccharified cassava flour broth was transferred to shake flasks fitted with air locks and autoclaved at 121 °C for 15 min for sterilization. For inoculum preparation, the yeast *Saccharomyces cerevisiae* (strain CA1185) was pitched in liquid YPD medium (20 g L^−1^ glucose, 20 g L^−1^ peptone, and 10 g L^−1^ yeast extract) and incubated at 28 °C with orbital agitation of 180 rpm until maximum growth. Cells were then collected by centrifugation at 8500 rpm and 4 °C for 15 min, and resuspended in a sterile 9 g L^−1^ NaCl solution, to attain a 200 mg fresh yeast mL^−1^ concentration. Cassava broth was then pitched with the inoculum containing at a 5 mg fresh yeast mL^−1^ concentration, obtained by dilution of the previously collected yeast, following the procedure reported by Kelbert et al. in [30]. Alcoholic fermentations were conducted at 28 °C, with orbital agitation of 180 rpm, accompanied by profiling of CO_2_ production by periodical mass loss measurements. Finally, a secondary fermentation process was carried out to maturate the obtained alcoholic beverages. The process consisted of transferring the fermented already liquors without removing the yeast to sterile bottles, which were left in a dark room at 6 °C for 60 days.

### 3.4. Distillation

For the production of cassava spirits, the alcoholic fermented cassava products were centrifuged at 4000 rpm for 15 min, for removal of yeast lees. Fractional distillation was conducted using a heating blanket, and a glass Vigreux column connected to a straight condenser with a distillation head, where a thermometer allowed controlling the vapor temperature. The temperature in the condenser was maintained at 10 °C employing a cooling bath in a closed circuit. Condensed fractions of fixed volume were collected at 76 °C, and then, the ethanol concentration was quantified by HPLC. After characterization, the obtained fractions were mixed to achieve the desired ethanol concentration, which was rectified to 40% (*v*/*v*) using ultrapure water from a Milli-Q System (Millipore Inc., Burlington, Massachusetts, USA).

### 3.5. Contact with Wood

After distillation, cassava spirits were divided into two parts: One remained without further processing, and the other was put in contact with oak chips for rapid induction of wood aromas. For this purpose, American oak cubes, 3 mm side, M+ toast (Seguin Moreau, Cognac, France) were immersed in the cassava spirit at a concentration of 20 g L^−1^. Extraction was performed during 48 h at 40 °C in anaerobic flasks with rubber stoppers and aluminum seals with orbital agitation of 200 min^−1^, following the proposed in previous works [16]. After contact, woods were separated from the spirits by decantation, which were further centrifuged for removal of suspended solids. 

### 3.6. Quantification of Fermentable Sugars and Ethanol

Carbohydrates were quantified by High-Performance Liquid Chromatography (HPLC), in agreement with the OIV method OIV-MA-BS-11 [31]. An Aminex HPX-87H (BioRad, Berkeley, California, USA) column was used and maintained at 60 °C. The eluent used was 5 mM H_2_SO_4_, at an isocratic flow of 0.6 mL min^−1^ and 20 µL of sample injection. Compounds were analyzed using a RI detector (Knauer, Berlin, Germany) and quantified with calibration curves prepared from pure standards. Quantification of sugars and ethanol was performed through Star-Chromatography Workstation (Varian, Walnut Creek, California, USA).

### 3.7. Analysis of Major Volatile Compounds

Major volatiles concentration was quantified in agreement with the OIV method OIV-MA-BS-14 [32], using a CP-9000 system (Chrompack, Middelburg, Netherlands) with a Meta-WAX capillary column (30 m × 0.25 mm × 0.25 µm) equipped with a flame ionization detector (FID), using helium as carrier gas with a flow rate of 1 mL min^−1^. Samples were diluted at a 1:5 factor, mixed with 4-nonanol as internal standard at a final concentration of 60 mg L^−1^ and 1 µL of sample was injected. The temperature of the injector and detector were maintained at 250 °C. The column was initially at 50 °C, heated to 177.5 °C at a 5 °C min^-1^ rate and then heated to 230 °C at 10 °C min^−1^, which was held for 15 min. Quantification was performed using software Star-Chromatography Workstation version 6.41 (Varian) supported by response factors and retention times determined with pure standards.

### 3.8. Analysis of Minor Volatile Compounds

Minor volatiles were analyzed by GC–MS following the previously validated method Coelho et al. 2020 [33]. Briefly, 8 mL of diluted samples (1:4 dilution factor) was extracted with 400 µL of dichloromethane (SupraSolv), with 4-nonanol as internal standard. A gas chromatograph Varian 3800 with a 1079 injector and an ion-trap mass spectrometer Varian Saturn 2000 was used. One microliter injections were made in splitless mode (30 s) in a Sapiens-Wax MS column (30 m; 0.15 mm; 0.15 µm film thickness, Teknokroma, Barcelona, Spain). The carrier gas was helium 49 (Praxair, Maia, Portugal) at a constant 1.3 mL min^−1^ flow. The detector was set to electronic impact mode with ionization energy of 70 eV, a mass acquisition range from 35 to 260 *m*/*z*, and 610 ms acquisition interval. The oven temperature was initially set to 60 °C for 2 min and then raised from 60 to 234 °C at a rate of 3 °C min^−1^, raised from 234 to 260 °C at 5 °C min^−1^, and finally maintained at 260 °C for 10 min. The injector temperature was 250 °C with a 30 mL min^−1^ split flow. Compounds were identified using MS Workstation version 6.9 (Varian) software, by comparing mass spectra and retention indices with those of pure standards and quantified as 4-nonanol equivalents.

### 3.9. Sensory Analysis

Two cassava distillates with replicates were evaluated by sensory analysis by six trained panelists from the official panel of Geographic Indication Protected of the Spirits and Traditional Liqueurs from Galicia (Spain). All the judges were experienced spirit tasters and all of them have previously participated in similar studies. Sensory analysis was performed in a professional standard room in agreement with the ISO Norm 8589 [34]. The evaluation was carried out using the QDA methodology [35] in order to establish the descriptors of the distillates. A constant sample volume of 30 mL of each spirit was evaluated in spirit-taster glasses at 12 °C. During the analysis, the judges smelled and tasted the samples, and the perceived descriptors were indicated. Then, they scored the intensity of each attribute using a 9-point scale, where 9 indicated a very high intensity. The relative frequency (*F*), relative intensity (*I*), and geometric mean (*GM*) of the different descriptors were calculated for each spirit. *GM* was calculated as the square root of the product between *I* and *F*, i.e., *GM*(%)= √(*I*×*F*)×100 where *I* corresponds to the sum of the intensities given by the panel for a descriptor, divided by the maximum possible intensity for this descriptor, and *F* is the number of times that the descriptor was mentioned divided by the maximum number of times that it could be mentioned. The descriptors were classified for each spirit by using the *GM* according to the International Organization for Standardization—ISO Norm 11035 [36], which made it possible to eliminate the descriptors whose geometric means were relatively low. This method allowed taking into account descriptors which were rarely mentioned but which were very important in terms of the perceived intensity, and descriptors with a low perceived intensity but which are mentioned often [37].

## 4. Conclusions

The use of enzymes for liquefaction and saccharification of cassava starch allows the production of a fermentable broth, which can be fermented for the production of a cassava alcoholic product with adequate ethanol concentration for distillation. The volatile fraction of cassava spirit was mainly composed by compounds produced by yeast during alcoholic fermentation. The spirit produced solely from cassava was well accepted by trained panelists. The application of oak chips allows further modification of cassava spirit sensory properties, allowing the production of an also accepted distilled beverage. Cassava is, therefore, a suitable raw material for the production of distilled spirits, using easily implementable biotechnological tools, being a feasible strategy for attaining added-value compounds from this crop.

## Figures and Tables

**Table 1:** Concentration (*C*) of major volatile compounds in cassava spirits, along with the reported sensory descriptors and the corresponding perception thresholds (*PT*), reported in [23]. Results represent the mean ± standard deviation of independent samples (n = 2).

	Cassava Spirit	Cassava Spirit + Oak Chips	*PT*	Descriptor
	*C* (mg L^−1^)	*C* (mg L^−1^)	*C* (mg L^−1^)	
acetaldehyde	39.2 ± 25.6	44.0 ± 33.1	10	Fresh, green
ethyl acetate	101.0 ± 55.2	124.6 ± 73.3	7.5	Solvent, fruity
methanol	23.7 ± 1.7	24.6 ± 0.5		
1-propanol	22.1 ± 4.7	24.3 ± 5.3	830	
2-methyl-1-propanol	88.1 ± 5.0	101.6 ± 14.1	40;75	Malty
2-methyl-1-butanol	77.0 ± 4.3	85.9 ± 7.9	7;30	Malty, solvent
3-methyl-1-butanol	478.4 ± 15.9	522.6 ± 37.6	7;30	Malty
2-phenylethanol	24.5 ± 3.1	27.0 ± 3.2	7.5;10	Flowery

**Table 2:** Concentration (*C*), linear retention index (*LRI*), and identification ions (*II*) of minor volatile compounds in cassava spirits, along with the reported sensory descriptors and the corresponding perception thresholds (*PT*) reported in [23]. Results represent the mean ± standard deviation of independent samples (n = 2).

Compound	*LRI*	Cassava Spirit	Cassava Spirit+ Oak Chips	*II*	*PT*	Descriptor
		*C* (µg L^−1^)	*C* (µg L^−1^)	(*m*/*z*)		
Esters						
isoamyl acetate	1119	1605.5 ± 30.6	1451.1 ± 229.3	43 + 55 + 70	30	Banana
ethyl hexanoate	1229	863.1 ± 26.9	848.3 ± 40.4	43 + 88 + 145	5	Fruity, Green Apple
ethyl octanoate	1429	350.9 ± 9.1	398.8 ± 15.7	55 + 88 + 127	2; 26	Apple, fruity, sweet
diethyl succinate	1668	-	33.3 ± 8.5	101 + 129	100,000	
2-phenylethyl acetate	1801	335.4 ± 9.8	249.3 ± 14.3	43 + 104	250	Flowery, sweet
ethyl hexadecanoate	2249	488.8 ± 35.6	80.6 ± 13.4	55 + 101 + 157 + 241 + 284		
Alcohols						
1-hexanol	1344	172.1 ± 1.1	179.1 ± 2.1	56 + 69	500; 2500	
Furan compounds						
furfural	1471	413.0 ± 53.0	1381.4 ± 37.3	39 + 95	8000	Smoky, almond
5-methylfurfural	1564	-	207.8 ± 14.1	53 + 109		
Acids						
hexanoic acid	1851	215.4 ± 25.9	406.8 ± 36.9	60 + 99	3000;8000	Fatty acids, vegetable oil
octanoic acid	2065	351.8 ± 28.7	438.2 ± 27.8	60 + 101	8800; 10,000	Fatty acids, vegetable oil
Phenolic compounds						
guaiacol	1848	-	79.5 ± 22.4	81 + 109 + 124	5	Smoky, almond
eugenol	2150	-	40.3 ± 4.6	77 + 103 + 164	7	Spicy, clove
2,6-dimethoxyphenol	2249	-	293.9 ± 16.3	65 + 93 + 154		
Lactones						
***cis***-oak lactone	1870	-	944.4 ± 3.7	41 + 71 + 99	20	Oak, wood
***trans***-oak lactone	1938	-	378.2 ± 16.2	41 + 69 + 99	20	
***γ***-nonalactone	2009	1038.0 ± 24.1	1193.2 ± 221.2	85		
***γ*** -decalactone	2122	40.6 ± 0.3	63.1 ± 4.4	85	5; 10	Fruity, peach
Aldehydes						
5-acetoxymethyl-2-furaldehyde	2183	-	130.6 ± 16.3	43 + 79 + 126		
vanillin	2543	-	566.3 ± 10.3	151	100	Vanilla, spicy
syringaldehyde	2929	-	4419.4 ± 60.0	182		
sinapaldehyde	3510	-	10009.3 ± 1072.4	45 + 165 + 208		

## References

[1] Liu Q., Liu J., Zhang P., He S. (2014). Root and Tuber Crops. Encycl. Agric. Food Syst..

[2] Parmar A., Sturm B., Hensel O. (2017). Crops that feed the world: Production and improvement of cassava for food, feed, and industrial uses. Food Secur..

[3] Uchechukwu-Agua A.D., Caleb O.J., Opara U.L. (2015). Postharvest Handling and Storage of Fresh Cassava Root and Products: a Review. Food Bioprocess Technol..

[4] Jakrawatana N., Pingmuangleka P., Gheewala S.H. (2016). Material flow management and cleaner production of cassava processing for future food, feed and fuel in Thailand. J. Clean. Prod..

[5] Alene A.D., Abdoulaye T., Rusike J., Labarta R., Creamer B., del Río M., Ceballos H., Becerra L.A. (2018). Identifying crop research priorities based on potential economic and poverty reduction impacts: The case of cassava in Africa, Asia, and Latin America. PLoS ONE.

[6] Enete A., Igbokwe E. (2009). Cassava Market Participation Decisions of Producing Households in Africa. Tropicultura.

[7] Colehour A.M., Meadow J.F., Liebert M.A., Cepon-Robins T.J., Gildner T.E., Urlacher S.S., Bohannan B.J.M., Snodgrass J.J., Sugiyama L.S. (2014). Local domestication of lactic acid bacteria via cassava beer fermentation. PeerJ.

[8] Das A.J., Seth D., Miyaji T., Deka S.C. (2015). Fermentation optimization for a probiotic local northeastern Indian rice beer and application to local cassava and plantain beer production. J. Inst. Brew..

[9] Ray R.C., Sivakumar P.S. (2009). Traditional and novel fermented foods and beverages from tropical root and tuber crops: review. Int. J. Food Sci. Technol..

[10] Kubo R., Funakawa S., Araki S., Kitabatake N. (2014). Production of indigenous alcoholic beverages in a rural village of Cameroon. J. Inst. Brew..

[11] Brito V.H. dos S., Cereda M.P. (2017). Fermented Foods and Beverages from Cassava (Manihot esculenta Crantz) in South America: Abstract.

[12] Lyumugabe F., Songa E.B. (2019). Traditional Fermented Alcoholic Beverages of Rwanda (Ikigage, Urwagwa, and Kanyanga): Production and Preservation.

[13] Zhang M., Xie L., Yin Z., Khanal S.K., Zhou Q. (2016). Biorefinery approach for cassava-based industrial wastes: Current status and opportunities. Bioresour. Technol..

[14] Mayer F.D., Gasparotto J.M., Klauck E., Werle L.B., Jahn S.L., Hoffmann R., Mazutti M.A. (2015). Conversion of cassava starch to ethanol and a byproduct under different hydrolysis conditions. Starch/Staerke.

[15] Ruiz M.I., Sanchez C.I., Torrresa R.G., Molina D.R. (2011). Enzymatic hydrolysis of cassava starch for production of bioethanol with a colombian wild yeast strain. J. Braz. Chem. Soc..

[16] Coelho E., Teixeira J.A., Domingues L., Tavares T., Oliveira J.M. (2019). Factors affecting extraction of adsorbed wine volatile compounds and wood extractives from used oak wood. Food Chem..

[17] Christoph N., Bauer-Christoph C. (2007). Flavour of spirit drinks: Raw materials, fermentation, distillation, and ageing. Flavours and Fragrances: Chemistry, Bioprocessing and Sustainability.

[18] Hazelwood L.A., Daran J.M., Van Maris A.J.A., Pronk J.T., Dickinson J.R. (2008). The Ehrlich pathway for fusel alcohol production: A century of research on Saccharomyces cerevisiae metabolism. Appl. Environ. Microbiol..

[19] Nascimento E.S.P., Cardoso D.R., Franco D.W. (2008). Quantitative Ester Analysis in Cachaça and Distilled Spirits by Gas Chromatography−Mass Spectrometry (GC−MS). J. Agric. Food Chem..

[20] Coelho E., Domingues L., Teixeira J.A., Oliveira J.M., Tavares T. (2019). Understanding wine sorption by oak wood: Modeling of wine uptake and characterization of volatile compounds retention. Food Res. Int..

[21] Cadahía E., Fernández de Simón B., Jalocha J. (2003). Volatile compounds in Spanish, French, and American oak woods after natural seasoning and toasting. J. Agric. Food Chem..

[22] Silva R., Aguiar T.Q., Coelho E., Jiménez A., Revuelta J.L., Domingues L. (2019). Metabolic engineering of Ashbya gossypii for deciphering the de novo biosynthesis of γ-lactones. Microb. Cell Fact..

[23] Romero-Guido C., Belo I., Ta T.M.N., Cao-Hoang L., Alchihab M., Gomes N., Thonart P., Teixeira J.A., Destain J., Waché Y. (2011). Biochemistry of lactone formation in yeast and fungi and its utilisation for the production of flavour and fragrance compounds. Appl. Microbiol. Biotechnol..

[24] Lee S.M., Lim H.J., Chang J.W., Hurh B.S., Kim Y.S. (2018). Investigation on the formations of volatile compounds, fatty acids, and γ-lactones in white and brown rice during fermentation. Food Chem..

[25] Lyu J., Nam P.W., Lee S.J., Lee K.G. (2013). Volatile compounds isolated from rice beers brewed with three medicinal plants. J. Inst. Brew..

[26] Marconi O., Sileoni V., Ceccaroni D., Perretti G. (2017). The Use of Rice in Brewing. Adv. Int. Rice Res..

[27] Poisson L., Schieberle P. (2008). Characterization of the most odor-active compounds in an American Bourbon whisky by application of the aroma extract dilution analysis. J. Agric. Food Chem..

[28] Caldeira I., Santos R., Ricardo-Da-Silva J.M., Anjos O., Mira H., Belchior A.P., Canas S. (2016). Kinetics of odorant compounds in wine brandies aged in different systems. Food Chem..

[29] Zhang B., Cai J., Duan C.Q., Reeves M.J., He F. (2015). A review of polyphenolics in oak woods. Int. J. Mol. Sci..

[30] Kelbert M., Romaní A., Coelho E., Pereira F.B., Teixeira J.A., Domingues L. (2016). Simultaneous Saccharification and Fermentation of Hydrothermal Pretreated Lignocellulosic Biomass: Evaluation of Process Performance Under Multiple Stress Conditions. Bioenergy Res..

[31] International Organisation of Vine and Wine (2019). Determination of sugars in spirit drinks of viti-vinicultural origin (OIV-MA-BS-11). Compendium of International Methods of Analysis of Spirituous Beverages of Vitivinicultural Origin.

[32] International Organisation of Vine and Wine (2019). Determination of the principal volatile substances of spirit drinks of viti-vinicultural origin (OIV-MA-BS-14). Compendium of International Methods of Analysis of Spirituous Beverages of Vitivinicultural Origin.

[33] Coelho E., Lemos M., Genisheva Z., Domingues L., Vilanova M., Oliveira J.M. (2020). Validation of a LLME/GC-MS methodology for quantification of volatile compounds in fermented beverages. Molecules.

[34] International Organization for Standardization (2007). Sensory Analysis-General Guidance for the Design of Test Rooms (ISO 8589:2007).

[35] Lawless H.T. (1998). Heymann Sensory Evaluation of Food Principles and Practices Second Edition.

[36] International Organization for Standardization (1994). Sensory Analysis-Identification and Selection of Descriptors for Establishing a Sensory Profile by a Multidimensional approach (ISO 11035:1994).

[37] Dravnieks A., Bock F.C., Powers J.J., Tibbetts M., Ford M. (1978). Comparison of odors directly and through profiling. Chem. Senses.

